# Progress in Ophthalmology

**Published:** 1900-12-01

**Authors:** 


### Progress in Ophthalmology.

Ranney,[1] of New York, in an article on "Does 'Cross Eye' Affect General Health," points out that it is an established and indisputable fact that eye strain ceases, and no leakage of nerve force takes place, whenever "cross eye" becomes an extreme deformity. The anxiety of intelligent parents whenever they bring a "cross-eyed' child to the oculist to have the eyes straightened is frequently much greater than the natural dread of an operation would account for, simply because their physician has told them that "such extreme 'cross eye' would eventually injure the health of the child" and that they must get rid of this source of danger. The correction of a marked eye deformity is unquestionably most desirable. It greatly improves the personal appearance, and it may add to the general comfort of the patient in the use of the eyes; but extreme degrees of "cross eye" (in which the two eyes cannot be made to hold single images) do not (save in exceptional cases) affect the general health, or justify correction as purely a therapeutical measure. The serious damage to health occurs in those who have two eyes that naturally tend to deviate, and perhaps occasionally may deviate for a time, from parallelism, but which can still be used together by an unconscious effort and a large expenditure of nerve force on the part of the patient. Writing of the delicate balance of the eyes so that single binocular vision may be maintained, he says:—In order to admit of the fusion of two visual images into one visual impression, the two eyes have to be accurately adjusted by four pairs of straight muscles and two pairs of oblique muscles, while the one pair of muscles that controls the upper eyelids acts in unison with the other six pairs, whenever the eyes are being adjusted so as to work properly together. All adjustments of the eyes, to insure the desired effects, have furthermore to be made with marvellous quickness and accuracy. The human mind can hardly grasp a numerical computation of these combinations of muscular effort or the lightning changes required in the seven pairs of muscles to keep the two eyes in perfect adjustment at all times and under all conditions. Imagine, for example, one driver (or even a band of drivers) trying to direct simultaneously, with seven pairs of reins, seven pairs of horses, through a series of most difficult and complicated evolutions at lightning speed. A slight mental conception may thus be formed of the complicated driving of the eyes; with the brain as the driver, and the eye muscles as the reins. One could hardly have a better description of the muscular arrangements of the eyes and it easily demonstrates that the general health may be upset by the tendency to, rather than by actual, squinting.

Sydney Stephenson,[2] in a paper on "The Place of Protargol in Eye Work," points out the limitations and disadvantages of solutions of silver nitrate. Nitrate of silver is not only precipitated by the albumen and chlorides of the plasma, but also by the discharges and secretions: *e.g.*, the sodium chloride of the tears. The local staining, after continued use, is indelible, the silver being deposited as fine granules in the lymphatic and perilymphatic structures. Experiments with protargol were conducted in three classes of cases—(1) blepharitis; (2) conjunctival, acute and chronic; (3) lachrymal. In the first class he thinks the application of ointment 10 per cent. possesses no advantage over the mercurial ointments that one is in the habit of using; but if these fail it may be tried. In gonorrhœal conjunctivitis, protargol used in solutions of from 10 to 50 per cent. gives better results than any other silver salt. The presence of corneal ulcers is no bar to its use, and, indeed, calls for more energetic treatment. In conjunctivitis due to the Kock-Weeks bacillus, 10 to 20 per cent. solutions are very good. In diplo-bacillary conjunctivitis protargol is distinctly inferior to sulphate or chloride of zinc. For trachoma the results are not satisfactory. Protargol in 5 to 10 per cent. solutions is the best agent for washing out the lachrymal sac in dacrysystitis.

Grossman and Loewenthal[3] report an epidemic of follicular conjunctivitis in a Liverpool school. Out of 720 children 420 were affected. In none of the cases was the pre-auricular gland enlarged—a point on which some authors lay great stress. It is not yet determined wherein lies the infectiousness of the discharge. Prognosis is good, and no scars result. The differential diagnosis is chiefly from vernal catarrh and granular conjunctivitis. The majority of slight cases were among the girls; the medium cases were equally divided; but there was a preponderance of the worst cases among the boys.

Mr. Sydney Stephenson[4] reports a case of rodent cancer of the cornea. He says it is undoubtedly one of the rarest affections of the eye. The patient, a female aged 60, was first seen by him in 1897; two months later the conditions were—a large, somewhat elevated patch of episcleritis lay on the outer side of eyeball, its central parts being rusty and soft-looking and its periphery pinkish red. The nodule was not tender. There was a little photophobia, but tension was normal and there was no iritis. The lower outer quadrant of the cornea was hazy, and a few vessels ran into it from the neighbouring sclero-corneal margin. There had been some pain, chiefly nocturnal, in the right temple. Two months later the outer side of the eyeball showed a large patch, the centre of which was slate-coloured and the periphery dull red. Its central, dark-coloured area looked almost pulpy in consistence. Neither tenderness nor pain existed. Patient was not seen again for eight months; the episcleritis had made satisfactory progress till a month before, when the cornea was slightly injured whilst removing her glasses. The eye since that time had been sensitive to light, had watered, and was generally uncomfortable. The episcleritis had left behind it a slightly depressed, lenticular patch of slaty hue. An oval ulcer of singular appearance now involved a limited portion of the outer side of the cornea below the horizontal meridian. Towards the sclera it was bounded by a vascular area and enlarged conjunctival vessels, and its cavity appeared to be filled with gelatinous material. The iris was active and not discoloured. A month later the ulcer had increased in size; its advancing edge was irregular and small vessels were present at its base. There was no actual pain, though the eye felt uneasy from time to time. The lesion never encroached on the pupillary area. A definite diagnosis of rodent cancer was made. Under cocain the ulcer was thoroughly burnt with galvano-cautery, special attention being paid to the aggressive margin. The operation was of a somewhat delicate character, owing to the care which had to be exercised not to interfere with the clear cornea in front of the pupil; it was followed by pain, œdema, and ecchymosis of the eyelids, as well as by marked redness of the globe. A month later the lower half of the ulcer had cicatrised in a smooth and satisfactory way, but the remainder was uneven, rugose, and permeated by fine vessels. The corneal sensibility in both eyes was diminished. Two days after this the galvano-cautery was freely applied to the roughened portion of the cornea; then the ulcer completely healed, leaving vis. $\frac{6}{18}$. The patient died a year later from influenza, but the corneal mischief had never recurred, nor did the eyes cause further trouble.

[1] Med. Rec., July 21, 1900. [2] Med. Chron., July, 1900. [3] Med. Chron., July, 1900. [4] Lancet, July 21, 1900.

